# Deprescription of Psychotropics in Children and Adolescents: Systematic Review of Guidelines and Development of a Deprescribing Algorithm

**DOI:** 10.1111/bcpt.70278

**Published:** 2026-07-24

**Authors:** Paul‐Benoît Fargier, Mark Horowitz, Rodolphe Charles, Valérie Rousselon, Erik Fakra, Marie‐Noëlle Beyens, Marie‐Laure Laroche

**Affiliations:** ^1^ Pharmacovigilance Centre Saint‐Étienne University Hospital Saint‐Étienne France; ^2^ Inserm Clinical Investigation Center (CIC‐EC 1408), North Hospital, Saint‐Étienne University Hospital Saint‐Étienne France; ^3^ Research and Development Department North East London NHS Foundation Trust Ilford UK; ^4^ Jacques‐Lisfranc Faculty of Medicine, Department of General Practice, Health and Innovation Campus Jean Monnet University Saint‐Priest‐en‐Jarez France; ^5^ Department of Child and Adolescent Psychopathology, Loire Autism Assessment Unit Saint‐Étienne University Hospital Saint‐Étienne France; ^6^ Department of Psychiatry Saint‐Étienne University Hospital Saint‐Étienne France; ^7^ Claude Bernard University Lyon 1, CNRS, INSERM, Lyon Neuroscience Research Center (CRNL), U1028 UMR 5292, PSYR2 Bron France; ^8^ Centre of Pharmacovigilance and Pharmacoepidemiology Pharmacology, Toxicology and Pharmacovigilance Unit Limoges University Hospital Limoges France; ^9^ UR 24134 (VieSante), IFR OMEGA HEALTH, Limoges University Limoges France

**Keywords:** clinical practice guidelines, deprescribing, paediatrics, psychotropic medication, systematic review

## Abstract

**Background:**

Psychotropics are increasingly prescribed in paediatrics despite limited evidence regarding their benefits and effectiveness. Although deprescribing approaches are established in adults, structured paediatric strategies remain poorly defined.

**Aims:**

To identify and evaluate clinical practice guidelines (CPGs) on psychotropic deprescribing in youth, to describe and discuss recommendations regarding when and how to deprescribe in practice.

**Methods:**

We systematically searched PubMed, Embase, PsycInfo, Scopus, Web of Science and grey literature (2015–2025). Two reviewers independently screened and evaluated the quality of the guidelines (AGREE II). We derived from this a psychotropic deprescribing algorithm in youth, supplemented with clinical expertise.

**Results:**

Among the 1390 records identified, none strictly met the methodological criteria for formal CPGs. Nevertheless, we assessed two papers that provided practical deprescribing recommendations and shared several characteristics with CPGs. One systematic review focused on antidepressant discontinuation, recommending tapering after remission with gradual dose reduction and monitoring to distinguish withdrawal from relapse. One narrative review provided child‐specific guidance for stimulants, recommending reassessment and possible discontinuation after 1 year of stability. Both were rated as ‘moderate’ using the AGREE II tool and provided limited practical detail on stepwise deprescribing procedures.

**Conclusion:**

Critical gap and urgent need for paediatric‐specific deprescribing guidelines. We propose an expert‐informed stepwise deprescribing algorithm.

## Plain Language Summary

Psychotropic medications are increasingly prescribed to children and adolescents, even though their long‐term benefits and harms are still uncertain. We searched major medical databases (2015–2025) for clear, practical guidance on how to safely reduce or stop these medicines in young people. Out of 1390 records, none strictly met the methodological criteria for formal clinical practice guidelines (CPGs). Nevertheless, two papers provided practical deprescribing recommendations and shared several characteristics with CPGs. One addressed antidepressant withdrawal, the other mainly concerned stimulants. Both offered only limited, sometimes vague advice on when and how to taper, and little help for everyday clinical decisions. No guidance was found for other drug classes, revealing a major gap in paediatric care.

## 1 Introduction

Psychotropics are frequently used in children and adolescents with mental disorders. The use of antidepressants and other drugs prescribed for attention‐deficit/hyperactivity disorder (ADHD) has risen sharply [1], and they may be used for the acute management of symptoms and behavioural crises associated with mental disorders, including agitation and aggression [2].

However, there is significant debate about the effectiveness of these medications, with, for example, a Cochrane review [3] finding that antidepressants have small and insignificant benefits in paediatric depression, bringing into question their use at all; and another Cochrane review showing that there is little evidence that antidepressants are more effective than psychotherapy [4]. Furthermore, there are significant harms to use of psychiatric medications in children, including stunted growth, emotional and sexual numbing, weight disturbance, type 2 diabetes and impairment of memory, concentration and sleep [5, 6, 7]. Many guidelines recommend that psychotropics should be combined with non‐pharmacological interventions (cognitive behavioural therapy—CBT, psychoeducation) [8, 9]. Unfortunately, access to non‐pharmacological approaches is not always possible because of barriers, including limited availability of providers and resources (long waiting lists with few opportunities for consultation and follow‐up), insufficient training in deprescribing among primary care clinicians, parental and/or child fears and financial restrictions [10, 11]. As a result, pharmacological treatments may be continued for prolonged periods in the absence of a clearly defined therapeutic indication or even before a formal psychiatric diagnosis has been properly established. In a nationwide Finnish prescription‐register cohort including 20 932 youths (1–17 years) who initiated a second‐generation antipsychotic between 2008 and 2016, the mean and median treatment durations were 509 days (95% CI, 500–517) and 317 days (95% CI, 306–325), respectively, with most prescriptions being off‐label [12]. In a population‐based Australian cohort [5] including 44 366 children and adolescents aged 5–18 years who initiated an antidepressant between 2014 and 2022, 33.0% remained persistent users at 1 year and 19.8% at 2 years, with persistence defined as continuous antidepressant supply without gaps exceeding 90 days between dispensing. Persistent use was more frequent among younger children, females and those receiving concomitant psychostimulants or antipsychotics, and increased over time.

Deprescribing is defined as ‘adjusting medications down to the minimum effective dosage or stopping them when the medication burden or potential for harm outweighs the benefit of the medication’ [13]. Deprescribing is one component of good prescribing. The goal is to use the lowest effective dose with the least number of medications and is not limited to medication cessation, though this is one potential outcome. Several reasons may lead clinicians to reduce or discontinue a psychotropic, including the occurrence of adverse drug reactions (ADRs) such as laboratory abnormalities, metabolic or cardiovascular complications, and psychological or behavioural adverse effects, which are more frequent in children [14]; as well as potential drug–drug interactions with concomitant treatments. Other factors include insufficient or waning effectiveness over time, an unclear therapeutic indication, parental and/or child concerns about uncertain long‐term effects, improvements in the child's living environment, routine review of medication burden or recovery.

However, there is a lack of guidance on how to safely taper psychotropics and minimize withdrawal symptoms in the paediatric population. Existing guidelines focus on adults and generally recommend reducing psychotropics over a 4‐week period. This duration was considered reasonable by expert consensus, based on the assumption that withdrawal symptoms were usually mild and lasted 1 or 2 weeks [15]. These guidelines were based on studies conducted by drug companies in the 1990s [16], focusing on people who had taken antidepressants for 8–12 weeks. More recent studies [17] have shown that withdrawal symptoms can last for months to years and have a substantial negative impact on patients' lives [18]. Withdrawal effects can be misdiagnosed as a relapse or new onset of a physical or psychiatric disorder [19] leading to the medication being prescribed again or a new one being added (‘prescribing cascade’). However, recent literature has increasingly questioned these empirical protocols, instead advocating alternative approaches, such as hyperbolic tapering of psychotropics in adults [20, 21]. Therefore, establishing clear guidelines for the reduction and discontinuation of psychotropics in young patients is of the utmost importance.

The primary aim of this review was to identify and evaluate the quality of national and international clinical practice guidelines (CPGs) relating to deprescription of psychotropics in children and adolescents, specifically relevant to clinical practice across all levels of healthcare settings. The secondary aim was to describe and discuss CPGs recommendations, including criteria for deciding when to deprescribe psychotropics, the process for stepwise tapering in order to limit withdrawal symptoms and recommended monitoring strategies. Finally, we proposed an expert‐informed stepwise psychotropic deprescribing algorithm in youth.

## 2 Methods

The protocol of this systematic review was registered on PROSPERO on 17 January 2025 (CRD42025629974), based on the methodological guide of Johnston et al. [22] and was reported according to the Preferred Reporting Items for Systematic Reviews and Meta‐Analyses (PRISMA) 2020 [23].

### 2.1 Search Strategy and Selection Criteria

A systematic search strategy was developed by the investigators in collaboration with medical librarians. Searches were conducted in PubMed MEDLINE, Embase, PsycInfo, Scopus and Web of Science for the period from 1 January 2015 to 3 February 2025. As deprescribing in children is a very recent field, we assumed that some relevant records might not be indexed with medical subject headings (MeSH) terms; therefore, grey literature was also explored through institutional website searches (e.g., websites of institutional bodies like the French National Authority for Health [Haute Autorité de Santé, HAS], the National Institute for Health and Care Excellence [NICE], the American Academy of Child and Adolescent Psychiatry [AACAP], the American Academy of Paediatrics [AAP], Google Scholar …). An additional search update was performed on 9 September 2025. The reference lists of all eligible reports were examined to identify any additional reports for potential inclusion.

Search terms included individual and combinations of terms such as deprescription, inappropriate prescribing, drug tapering, polypharmacy, dose reduction, withdrawal symptom, discontinuation, central nervous system agents, psychotropic drugs, antipsychotic agents, anticonvulsants (used as mood stabilizers), mood stabilizers, antidepressive agents, histamine antagonists, hypnotics and sedatives, melatonin, psychostimulant, mental health, child psychiatry, adolescent psychiatry, practice guidelines. Language was restricted to English and French. The search strategy was initially constructed using MeSH terms in PubMed and subsequently adapted for the other databases, with the support of medical librarians. The complete search strategy is available in Appendix 1.

CPGs were eligible for inclusion if: (i) they contained at least one eligible recommendation on deprescribing process (how to reduce, taper, discontinue, monitor or manage withdrawal symptoms) for psychotropics used in paediatric and adolescent mental health (including patients with neurodevelopmental disorders) such as antipsychotics, antidepressants, benzodiazepines, psychostimulants, mood stabilizers and melatonin; (ii) they were published or updated within the last 10 years and were the most updated version if multiple versions of CPGs existed; (iii) they were designed for healthcare providers (e.g., psychiatrists, paediatricians, general practitioners, pharmacists). Expert recommendations and expert opinions were also included.

CPGs were excluded if their content focused on other diagnoses (particularly comorbidities associated with neurodevelopmental disorders like epilepsy or metabolic syndrome) or other age groups. Letters to the editor, editorials, commentaries and conference abstracts not followed by full publications were excluded. Inclusion and exclusion criteria are summarized in Table 1.

**TABLE 1:** Inclusion and exclusion criteria.

Inclusion criteria
1. Scope: clinical practice guidelines focussing on the deprescribing or reduction of psychotropic medications in child and adolescent psychiatry (antipsychotics, antidepressants, benzodiazepines, psychostimulants, mood stabilizers and melatonin) 2. Recommendations: at least one eligible recommendation is reported (how to reduce, taper, discontinue, monitor or manage withdrawal symptoms) 3. Target audience: guidelines intended for healthcare providers (e.g., psychiatrists, paediatricians, general practitioners) 4. CPGs published from 1 January 2015 to 3 February 2025 5. Latest version only 6. Language: English or French 7. Publishing region: worldwide

Exclusion criteria
1. CPGs that focus on other age groups (> 18 years old) 2. CPGs primarily addressing other diseases (such as epilepsy, metabolic syndrome) 3. Letters to the editor, editorials, commentaries and conference abstracts not followed by full publications

### 2.2 Screening and Data Extraction

Results of the literature search were imported into Rayyan (https://www.rayyan.ai/), which was used for screening, deduplication and data extraction. Two reviewers (P‐B.F. and M‐L.L.) independently screened the titles and abstracts of all retrieved articles and assessed the full‐text records against the inclusion and exclusion criteria. Any discrepancies in assessment were resolved by a third reviewer (M‐N.B.). The following data were independently extracted by two reviewers (P‐B.F. and M‐L.L.): study characteristics (first author, year of publication, location, type of journal, study design, data source, population), exposure (drug class studied), physician's specialty, type of mental disorder(s) and recommendations (details of recommendations for discontinuation or stepwise process for tapering and limiting withdrawal symptoms and recommended monitoring strategies within guideline texts, tables, algorithms and/or decision pathways).

### 2.3 Strategy for Data Analysis

Guideline recommendations were independently synthesized by two reviewers (P‐B.F. and M‐L.L.), and the findings were compiled into tables.

### 2.4 Quality Assessment

Two reviewers (P‐B.F. and M‐L.L.) independently evaluated the quality of the included CPGs using the Appraisal of Guidelines for Research and Evaluation II (AGREE II) tool (https://www.agreetrust.org/resource‐centre/agree‐plus/) [24], which is a validated instrument to assess the methodological rigour and transparency of CPGs. It contains 23 items across six domains. The two investigators had to answer 23 questions for each guideline using a scale of 1 (*strongly disagree*) to 7 (*strongly agree*). Finally, the scores were summed, normalized to percentages and weighted according to the number of items in each domain to calculate an overall mean score. This score allowed the guidelines to be categorized by their overall quality: high quality (≥ 60%), moderate quality (30%–59%) or low quality (< 30%). Inter‐rater reliability between reviewers was evaluated using a two‐way random‐effects intraclass correlation coefficient (ICC) for absolute agreement, based on the mean of the two reviewers (ICC(2,2)) [25]. Any discrepancies were resolved through consensus (Appendix 2).

## 3 Results

**FIGURE 1:**
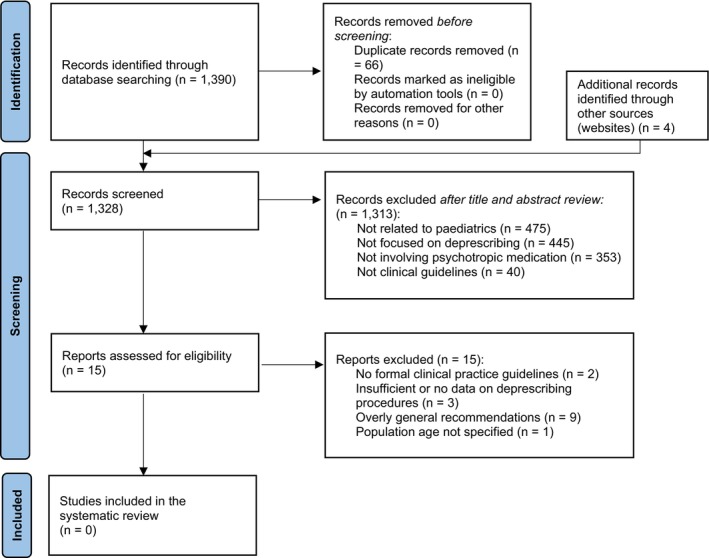
PRISMA 2020 flow diagram.

A total of 1390 records were identified through database searches, with four additional records retrieved from websites (Figure 1). After eliminating 66 duplicates, 1328 records remained for screening. Of these, 1313 were excluded after title and abstract review. Of the 15 full‐text reports screened, 13 were excluded: three for insufficient data on deprescribing (no recommendations on treatment withdrawal, tapering strategies, monitoring or the management of withdrawal symptoms; deprescribing only mentioned marginally, in one or two sentences, without structured or clinically applicable guidance), six for overly general recommendations and one for unclear age population. Finally, no papers were strictly identified as CPGs. However, two papers were retained for further analysis, consisting of one systematic review [26] and one narrative review [27], as the authors considered that the formulation and presentation of their results and recommendations closely resembled guideline recommendations, thereby allowing the application of the AGREE II tool. Both were judged to have ‘moderate’ methodological quality according to the AGREE II criteria. Notably, the scores for Domain 5 (applicability) were very low (14.6% for Stimpfl et al. [26] and 0% for Vinkers et al. [27]). Inter‐rater reliability across AGREE II domain scores was good (ICC(2,2) = 0.98). Further details of the quality assessment are outlined in Appendix 2.

The study by Stimpfl et al. [26] is a recent systematic review of antidepressant discontinuation in children and adolescents. Thirteen randomized controlled trials (RCTs) involving 3026 patients treated with different classes of antidepressants were analysed:

- selective serotonin reuptake inhibitors (SSRIs: fluoxetine, sertraline, paroxetine, citalopram, escitalopram, fluvoxamine and vilazodone);
- serotonin and noradrenaline reuptake inhibitors (SNRIs: venlafaxine, duloxetine, desvenlafaxine and levomilnacipran);
- tricyclic antidepressants (TCAs: imipramine, clomipramine and amitriptyline);
- atypical antidepressants (bupropion, mirtazapine and vortioxetine).

The authors provide molecule‐specific summary data for each molecule in the class of antidepressants in children and adolescents, but no actual dose‐tapering protocols. General recommendations proposed by the authors regarding antidepressant deprescribing (who to deprescribe, when to do it, and how to proceed) are summarized in Table 2.

**TABLE 2:** Criteria for antidepressant deprescribing in youth (according to Stimpfl et al.).

Who
• Children or teens who are much better or in remission, with no major daily difficulties. • Those who have troubling side effects or who would rather try non‐medication options. • Children whose families can take part in monitoring and decisions. • Not good candidates: youth with ongoing symptoms, high risk of relapse or important other medical/psychiatric problems.

When (best timing)
• After a stable period of improvement—usually 9–12 months after symptoms have gone, but sometimes longer (12 months or more) if the depression is chronic or has come back before. • Choose a calm period in life (e.g., summer break) and avoid times of stress like exams or big transitions. • If the patient is in or has finished therapy (like CBT or relapse‐prevention CBT), this can help reduce the risk of relapse when stopping.

How (how to taper safely)
• Individual plan: balance the possible benefits of stopping with the risks of relapse or withdrawal. Consider the child's health, treatment history, other medications, family and school situation. • Shared planning: discuss with the family the potential withdrawal effects, noting that their intensity may vary across different agents; and what signs of relapse to watch for. • Slow taper: gradually reduce the dose. If withdrawal symptoms occur, return to the previous dose and resume tapering at a slower pace. • Regular follow‐up: check symptoms and functioning often, involve parents and school if possible.

The study by Vinkers et al. [27], is a narrative review of psychotropics, with stimulants being the only class relevant to our review, the others concerning adults exclusively. The authors argue that about 30% of children with ADHD can discontinue stimulants without symptom worsening at 3 months. Current guidance recommends annual re‐evaluation of stimulant therapy, with discontinuation considered after ≥ 1 year of stability, in the absence of recent dose changes or prior relapse during drug holidays. They argue that stimulants can usually be stopped without tapering, although transient withdrawal symptoms may occur (fatigue, insomnia, hypersomnia, vivid dreaming, irritability, transient motor symptoms); structured drug holidays may reduce both symptoms and adverse effects. Short‐term benzodiazepines (≤ 2 weeks) may help manage irritability and sleep disturbances.

## 4 Discussion

### 4.1 Limitations of Existing Paediatric Psychotropic Deprescribing Guidelines

This study aimed to identify, evaluate, describe and discuss international CPGs on deprescribing psychotropics in children and adolescents, including withdrawal management, and to determine whether clear, evidence‐based recommendations for clinical practice exist.

Despite a broad search across multiple databases and institutional websites, no papers fully met our inclusion criteria. However, two papers [26, 27] were assessed because their content and presentation resembled CPGs; nevertheless, their methodological quality was judged to be moderate.

Indeed, they paid little attention to the most recent evidence [17, 18, 19, 20, 21, 28], which limits their clinical relevance. They provided no practical guidance on how to reduce the dose, what a ‘slow reduction’ means, or how to individualize the taper according to the patient's profile. There was no mention of protracted withdrawal, nor of severe manifestations such as akathisia or suicidality, and little guidance was provided regarding the monitoring of withdrawal symptoms, despite these issues being among the main challenges of the deprescribing process [29, 30, 31]. The paper by Vinkers [27] stated that stimulants could generally be discontinued without tapering; however, it lacked precision by failing to specify that this primarily applies to stimulant monotherapy, whereas much greater caution is required when stimulants are combined with antipsychotics because of the risk of acute dyskinesia [32].

These gaps in the literature led us to discuss withdrawal syndromes, tapering strategies and developmental considerations when considering psychotropic deprescribing in young people and to propose an expert‐informed stepwise deprescribing algorithm.

### 4.2 Withdrawal Syndromes, Tapering Strategies and Developmental Considerations

A recent pharmacovigilance study conducted in adults and adolescents aged ≥ 12 years reported that SSRIs were associated with a higher reporting rate of withdrawal syndromes compared with other drug classes [33]. The study also observed that withdrawal symptoms were more frequently severe in adolescents, males, individuals receiving polypharmacy and those treated for a longer period. The severity and clinical features of withdrawal may vary according to the class (Table 3), the dose and the half‐life of the drug [30]. For instance, the incidence of withdrawal symptoms appears to be high with paroxetine and moderate with sertraline and fluoxetine [33].

**TABLE 3:** Withdrawal symptoms (non‐exhaustive list).

Antidepressants	Nervous system: dizziness, light‐headedness, vertigo, tremor, ataxia, visual disturbances, akathisia Somatic: lethargy, fatigue, headache, sweating, myalgia, flu‐like symptoms, muscle cramps, tachycardia, hypertension Gastrointestinal: nausea, vomiting, diarrhoea, abdominal discomfort, abdominal cramps, abdominal distension Sensory: numbness, tingling, electric/shock‐like sensations, blurred vision, paraesthesia, pruritus, genital hypersensitivity Sleep‐disturbance: insomnia, vivid dreams, nightmares Psychological/affective: irritability, dysphoria, low mood, anxiety, nervousness, agitation, suicidality
Stimulants	Somatic: fatigue, headache, muscle cramps, vomiting, light sensitivity, weight gain Nervous system: acute dystonia, particularly when used concurrently with antipsychotics (aripiprazole, risperidone)
Antipsychotics	Serotonin withdrawal syndrome (i.e., flu‐like symptoms, sweating, chills; dizziness, light‐headedness, tachycardia; diarrhoea, loose stools, abdominal pain; restlessness, myalgia, rigidity, hyperreflexia, inducible clonus; paraesthesia, electric shock sensations, zaps; confusion, disorientation, amnesia, coma; premature ejaculation, genital hypersensitivity) Muscarinic cholinergic withdrawal syndrome (i.e., agitation, insomnia, anxiety, depression; dizziness, light‐headedness, tachycardia; nausea, vomiting, salivation, diarrhoea, loose stools, abdominal cramp; tremor, parkinsonism, restlessness, myalgia, rigidity, myosis; paraesthesia; confusion, disorientation; hypothermia, sweating, diaphoresis) Adrenergic withdrawal syndrome (i.e., headache, anxiety, agitation; increased blood pressure, increased heart rate, risk of myocardial infarction, angina pectoris, palpitations, chest pain; pre‐syncope, tremulousness; sweating) Histaminic withdrawal syndrome (i.e., irritability, depressed affect, insomnia, agitation; loss of appetite, nausea; tremulousness, incoordination, increased inducible seizure; lethargy, amnesia)
Benzodiazepines	Minor and frequent withdrawal symptoms: sweating, tachycardia, nausea, visual changes, tremor, confusion, restlessness, weakness, pain, malaise, itching, skin rash, postural hypotension, constipation, dry mouth, increased urinary frequency Major and rare withdrawal symptoms: psychosis, seizures (even in patients with no history of prior seizures)

Three types of withdrawal syndromes have been described in the adult literature [29, 31, 33, 34]:

- **New withdrawal symptoms**: Newly emerging symptoms for the patient, generally short‐lasting, transient and reversible. These can include features common to most psychotropics, as well as specific manifestations unique to certain drug classes (see Table 3);
- **Rebound symptoms**: Often transient and reversible reactions corresponding to a rapid return of the initial disorder's symptoms, often with greater intensity than before treatment;
- **Persistent post‐withdrawal disorders** (or ‘protracted withdrawal syndromes’): though they can onset later not just for fluoxetine, with its long half‐life, but also for short half‐life drugs, for reasons that are unclear. Long‐lasting, severe and sometimes potentially irreversible manifestations. They may include intensified rebound symptoms, novel withdrawal effects or even new‐onset disorders not present before treatment, persisting for more than 6 weeks after discontinuation.

Withdrawal symptoms encompass both psychological and physical dimensions. Psychological symptoms frequently include worsened mood, irritability, agitation, nervousness, confusion, insomnia, emotional lability and depersonalisation or derealisation. Physical symptoms may present as dizziness, headache, nausea, sweating, muscle aches, unsteady gait, paraesthesia or the characteristic ‘electric zaps’. Onset typically occurs within days of dose reduction or cessation—though they can onset later not just for fluoxetine, with its long half‐life, but also for short half‐life drugs, for reasons that are unclear. The course often follows a wave pattern (if tapering is performed in small steps such as 10% of the most recent dose): *(i) emergence, (ii) worsening, (iii) peak intensity, (iv) then gradual resolution (except for persistent post withdrawal disorders)*.

Distinguishing withdrawal symptoms from relapse is sometimes challenging [19, 29], because many psychological symptoms are shared. Several distinguishing features are helpful [19, 31, 34, 35]:

1. **Timing**: Withdrawal usually appears rapidly after dose reduction (hours to days), although it can be delayed by weeks or longer (possibly because downstream effects take time to reach a threshold); whereas relapse tends to develop later (weeks to months).
2. **Accompanying physical symptoms or symptoms not present in the underlying condition**: The presence of somatic features such as dizziness, nausea, sweating or neurological signs like ‘brain zaps’ strongly suggests withdrawal rather than relapse because such manifestations are atypical of primary depressive or anxiety disorders. However, psychological symptoms are the most common symptoms of withdrawal—these can be distinguished from symptoms of the underlying condition in terms of differences in quality or intensity.
3. **Response to reinstatement**: Withdrawal symptoms typically resolve quickly (within days) after reintroducing the medication if performed soon after cessation, although responses are more unpredictable if reinstatement is delayed; whereas relapse requires weeks to show improvement.

Misinterpretation of these manifestations between withdrawal symptoms and relapse may lead to premature reinstatement of the discontinued drug or to inappropriate therapeutic escalation, such as the addition of unnecessary medications. Moreover, some patients who wish to discontinue their medication may feel reluctant or anxious to proceed without appropriate supervision and structured support [36]. Clinicians and caregivers should therefore be trained to differentiate withdrawal phenomena from relapse to ensure accurate clinical decision‐making.

The review by Stimpfl et al. [26] suggests adding adjunctive medication to counterbalance withdrawal symptoms; however, this approach may be counterproductive, as it promotes the introduction of additional drugs, whereas the intended goal was to reduce or stop medication. In general, the emergence of withdrawal symptoms is best managed by slowing tapering rather than additional medications.

Furthermore, there were few explicit mentions of hyperbolic tapering [21, 30, 37] or formulation adjustments [38] in the reviewed recommendations. Most dose–response relationships in pharmacology assume a hyperbolic Emax‐type (Michaelis–Menten) curve [30]; hence, a fixed dose reduction will not have an identical effect throughout the range. Although receptor occupancy changes only slightly at high doses, an equivalent dose reduction produces a much larger effect at low doses [35]. Using citalopram as an example, a decrease from 20 to 15 mg will decrease serotonin transporter inhibition by 3% (absolute decrease), whereas a similar decrease from 5 to 0 mg will be associated with a 58% reduction [35]. As most drugs provoke neurobiological changes through changes in receptor sensitivity and density and through downstream signalling during chronic administration, the body adapts itself to sustain its new balance amidst continuous drug exposure [35]. A rapid or linear taper might suddenly disrupt this new balance and trigger withdrawal symptoms. The hyperbolic tapering method [20, 21, 30] recommends reducing doses according to the effect on target receptors instead of fixed milligramme amounts, with gradually reducing dose decrements as doses become smaller and allow for a smoother fall‐off of effect.

In children, developmental factors influence both pharmacokinetics (enzyme maturation, protein binding, distribution volume and clearance [39]) and pharmacodynamics (as receptor density peaks during the preschool years and gradually declines to adult levels in late adolescence [40]). These developmental variations may shift the position or slope of the dose–response curve, but not its overall hyperbolic shape. Thus, the same principles of hyperbolic tapering will apply to children and therefore, it is advisable to start with a small proportional reduction following the hyperbolic method, monitor the response of the child and adjust the tapering speed according to the patient's response. However, this proposal of a tapering dose strategy in paediatric care does not yet have randomized clinical evidence supporting its effectiveness. Finally, it should be noted that there is no randomized evidence that a linear 4‐week taper is effective, with many studies showing most adult patients cannot tolerate this approach [41]. Cohort studies in adults routinely find that hyperbolic tapering allows patients who were previously unable to discontinue their medication using traditional linear tapering to stop treatment more safely and with fewer adverse effects [42]; however, these findings remain based on studies without control groups.

### 4.3 Recommendations for Practice: From Evidence‐Based Recommendations to an Expert‐Informed Stepwise Deprescribing Algorithm

**FIGURE 2:**
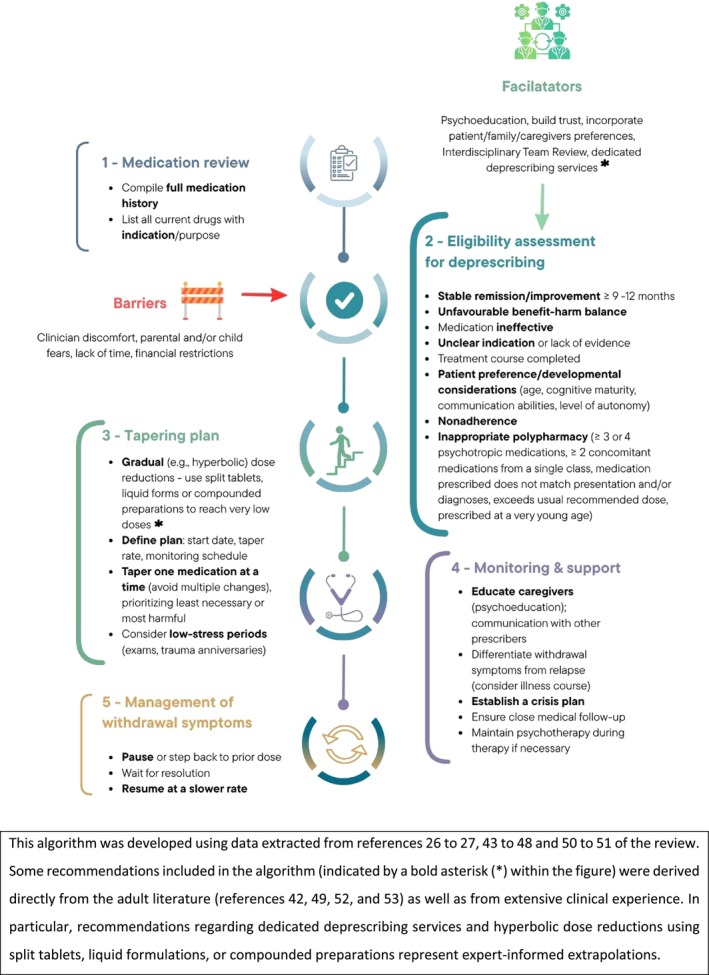
Expert‐informed algorithm for psychotropic deprescribing in children and adolescents.

The recommendations we identified were often ambiguous, occasionally raised safety concerns and were frequently difficult to translate into routine clinical practice. To help address the lack of practical guidance, we propose an expert‐informed stepwise algorithm for deprescribing (Figure 2), developed from the currently available literature, pharmacological plausibility, expert consensus and emerging clinical observations from both paediatric and adult populations. Although this algorithm may help structure psychotropic deprescribing in clinical practice, it should be considered preliminary and cannot replace a fully operational evidence‐based deprescribing framework or the need for future RCTs.

1 Medication review

First, a comprehensive medication review should include a full treatment history, a precise list of all current prescriptions with their corresponding indications, as well as the date of initiation and the clinical context in which each medication was introduced [32, 43, 44, 45].

2 Eligibility assessment for deprescribing

Eligibility for deprescribing should be carefully assessed. Criteria in favour of deprescribing [32, 43, 44, 45, 46, 47] may be: (i) a stable remission or clinical improvement over a period of 9–12 months; (ii) an unfavourable benefit‐harm balance; (iii) a lack of a valid indication, or the natural completion of a treatment course; (iv) non‐adherence, low treatment satisfaction, inappropriate polypharmacy (for instance, two or more psychotropics from the same class), prescriptions that no longer match the patient's presentation, or medications given at unusually young ages or at doses above standard recommendations.

**The timing of deprescribing** should be taken into account: for instance, choosing periods of lower stress, avoiding examinations, anniversaries of trauma or other potentially destabilizing events may be beneficial for some individuals [44, 45, 47]. However, slower tapering may generally reduce the need for such completely stress‐free periods.

**Developmental considerations** should also be taken into account [48]. The child's age, cognitive maturity, communication abilities and level of autonomy may influence both the decision to deprescribe and the way the process is implemented. Very young children, or children with neurodevelopmental disorders, may have difficulty recognizing, verbalizing, or differentiating adverse effects, withdrawal symptoms, relapse or ordinary developmental fluctuations, which may therefore necessitate close caregiver or professional involvement for monitoring during the process. In contrast, older children and adolescents may be able to express their own treatment preferences and concerns and should be involved in shared decision‐making whenever appropriate. Practical aspects should also be anticipated, including the child's daily routine, school attendance and the need for medication administration by parents, caregivers or school staff during the tapering process.

3 Tapering plan

A structured tapering plan should be developed, usually based on gradual dose reductions (e.g., hyperbolic reductions) tailored to the individual patient. When very low doses are required, tablet splitting or liquid formulations may be considered, although these options are not always available or suitable. In such cases, compounded or ‘off‐label’ preparations may be needed to enable smaller and more precise dose reductions [49].

The plan must specify a start date, tapering rate and monitoring strategy [32, 43, 44, 45, 47]. Medications should generally be tapered one at a time, beginning with the least useful or most harmful. If several agents with opposing sedative and stimulant properties are being discontinued, alternating dose reductions may help avoid abrupt changes in energy balance (e.g., reducing methylphenidate by 25%, then risperidone by 25% and continuing step by step until both are withdrawn) [32].

4 Monitoring and support

Close monitoring is essential. This includes caregiver education, continuous communication with other prescribers, careful differentiation between withdrawal symptoms and relapse (considering the natural course of illness) and the preparation of a crisis plan. Clinical support should remain active throughout the process. This requires regular medical follow‐up and, where appropriate, the continuation or reinforcement of psychotherapy.

5 Management of withdrawal symptoms

If withdrawal symptoms arise, the strategy is to pause or return to the previous dose, wait until resolution and then resume tapering at a slower pace [32, 43, 44, 45, 46, 47].

These five steps should involve a multidisciplinary team [50, 51], including physicians, pharmacists [52], nurses, psychologists, and occupational therapists, parents and caregivers at each stage, in order to integrate complementary expertise and ensure continuity of care. Dedicated deprescribing services could also be considered, as has already been instituted in adult populations [53].

### 4.4 Strengths and Limitations

A major strength of this work is that it provides a comprehensive and methodologically rigorous synthesis of the available recommendation‐oriented literature on paediatric psychotropic deprescribing, integrating the most recent evidence. All stages of the review were conducted in accordance with PRISMA standards and methodological guidance for systematic reviews of CPGs. Several limitations should nevertheless be acknowledged.

First, we encountered methodological challenges in designing the search strategy, as some potentially relevant records were not indexed in major bibliographic databases or were not associated with specific MeSH terms. Consequently, some relevant recommendations may have been missed despite the use of complementary searches through institutional websites and grey literature sources.

Second, the two analysed papers did not constitute formal CPGs in the strict methodological sense. Nevertheless, both provided structured, clinically oriented and practically applicable deprescribing recommendations that were considered sufficiently guideline‐like to warrant a focused analysis within this review. The systematic review by Stimpfl et al. provided detailed and operational recommendations regarding tapering strategies, cross‐titration approaches, pharmacokinetic considerations and withdrawal management, whereas the narrative review by Vinkers et al. was organized around clinically relevant deprescribing questions and proposed structured practical recommendations.

For these reasons, we considered AGREE II to be more appropriate than AMSTAR 2 while acknowledging the limitations of this methodological choice. AMSTAR 2 was considered for the systematic review by Stimpfl et al.; however, the methodological confidence would likely have been rated as critically low because several critical AMSTAR 2 domains were not fulfilled, including the absence of a reported protocol, no formal risk‐of‐bias assessment and no indication of duplicate study selection or data extraction. In this context, AGREE II was used pragmatically to appraise the recommendation‐oriented and clinically applicable dimensions of the included papers rather than to assess them as formal CPGs.

Finally, the restriction to English‐ and French‐language publications may have led to the exclusion of potentially relevant recommendations or guidance documents from other healthcare systems, particularly from countries such as Germany, the Netherlands or Spain, thereby limiting the international representativeness of the review. However, we believe that this bias is likely limited, as the development of national recommendations generally relies on published scientific evidence, which is predominantly available in English.

## 5 Conclusion

The principal finding of this review is the very limited availability and quality of deprescribing CPGs for psychotropics in child and adolescent mental health. We therefore propose a general algorithm for psychotropic deprescribing in these populations, based on the latest available evidence. Even in the absence of paediatric‐specific protocols, a hyperbolic tapering approach (involving very gradual dose reductions, particularly at lower dose ranges) appears to be a promising and pharmacologically plausible strategy, supported by cohort studies in adult populations, although its effectiveness in paediatric populations remains to be established in future studies. Future research should focus on generating stronger evidence to guide psychotropic deprescribing in children and adolescents. Priorities include randomized trials comparing different tapering strategies, prospective studies that carefully monitor withdrawal symptoms and relapse and the validation of paediatric‐specific deprescribing protocols and clinical guidelines. These studies are needed to support safer and more effective deprescribing in paediatric practice.

## Funding

The authors have nothing to report.

## Ethics Statement

The authors have nothing to report.

## Conflicts of Interest

This article received no specific grant from any funding agency, commercial or not‐for‐profit sectors. M.H. receives royalties from The Maudsley Deprescribing Guidelines. M.H. is a co‐founder and consultant to Outro Health, a US‐based digital clinic helping adults to stop no longer needed antidepressants, and has received consulting fees, lecture honoraria and conference travel reimbursements. M.H. holds a salaried Clinical Research Fellowship at North East London NHS Foundation Trust. M.H. is a co‐applicant on the Australian RELEASE and RELEASE+ trials, funded by the Medical Research Future Fund and the National Health and Medical Research Council, with no personal payments received.

## Data Availability

Data sharing is not applicable to this article as no datasets were generated or analysed during the current study.

## Appendix 1 Search Information

**Database requests**

1 Pubmed

(‘Mental Health’[MeSH] OR ‘Mental Disorders’[MeSH] OR ‘Psychiatry’[MeSH] OR ‘psychological well‐being’[tiab] OR ‘mental illness’[tiab] OR ‘psychiatric disorder’[tiab] OR ‘psychopathology’[tiab])

OR

(‘Psychotropic Drugs’[MeSH] OR ‘Psychotropic Medication’[tiab] OR ‘Central Nervous System Agents’[MeSH] OR ‘Antipsychotic Agents’[MeSH] OR ‘Antidepressants’[MeSH] OR ‘Central Nervous System Stimulants’[MeSH] OR ‘Psychostimulant’[tiab] OR ‘Anti‐Anxiety Agents’[MeSH] OR ‘Anxiolytic’[tiab] OR ‘Benzodiazepines’[MeSH] OR ‘Histamine Antagonists’[MeSH] OR ‘Antihistamine*’[tiab] OR ‘Hypnotics and Sedatives’[MeSH] OR ‘Sedative*’[tiab] OR ‘Anticonvulsants’[MeSH] OR ‘Melatonin’[MeSH] OR ‘Gabapentinoids’[tiab] OR ‘gamma‐Aminobutyric Acid’[tiab] OR ‘Mood Stabilizer*’[tiab])

AND

(‘Drug Deprescriptions’[All Fields] OR ‘Drug Therapy/methods’[MeSH] OR ‘Deprescription’[tiab] OR ‘Deprescribing’[tiab] OR ‘Tapering’[tiab] OR ‘Dose‐Reduction’[tiab] OR ‘Medication Withdrawal’[tiab] OR ‘Weaning’[tiab] OR ‘Stopping Medication’[tiab] OR ‘Medication Cessation’[tiab] OR ‘Drug Withdrawal’[tiab] OR ‘Substance Withdrawal Syndrome’[MeSH] OR ‘Withdrawal Syndrome’[tiab] OR ‘Inappropriate Prescribing’[MeSH] OR ‘Polypharmacy’[MeSH] OR ‘Medication Overuse’[tiab] OR ‘Multiple Medications’[tiab])

AND

(‘Practice Guidelines as Topic’[MeSH] OR ‘practice guideline’[MeSH] OR ‘Guideline Adherence’[MeSH] OR ‘Clinical Practice Guideline’[tiab] OR ‘Recommendation’[tiab] OR

‘Expert Opinion’[tiab] OR ‘Consensus’[tiab] OR ‘Best Practice*’[tiab] OR ‘Evidence‐Based Medicine’[MeSH] OR ‘Systematic Review*’[tiab])

NOT

(‘Adult’[Mesh])

2 Embase, PsycInfo, Scopus and Web of Science

The search strategy was initially constructed using MeSH terms in PubMed and subsequently adapted for the other databases, with the support of medical librarians.

**Grey literature keywords**

deprescription, inappropriate prescribing, drug tapering, polypharmacy, dose reduction, withdrawal symptom, discontinuation, central nervous system agents, psychotropic drugs, antipsychotic agents, anticonvulsants, mood stabilizers, antidepressive agents, histamine antagonists, hypnotics and sedatives, melatonin, psychostimulant, mental health, child psychiatry, adolescent psychiatry, practice guidelines

## Appendix 2 Quality Assessment (AGREE II) Methodology

Calculated domain score: Sum of scores for Reviewers 1 and 2 for each of the six domains.

Scaled domain score equation (%) : Calculate a scaled domain score for each domain using the following equation:

$$\frac{\text{Calculted domain score}-\text{Minimum possible domain score}}{\text{Maximum possible domain score}-\text{Minimum possible domain score}}\;x\;100$$

Maximum and minimum possible score for each domain for AGREE II:

**Table:**

	Items per domain	Maximum possible score	Minimum possible score
Domains 1, 2 and 4	3	42	6
Domain 3	8	112	16
Domain 5	4	56	8
Domain 6	2	28	4

Maximum possible score = 7 (*strongly agree*) × number of items per domain × 2 (reviewers)

Minimum possible score = 1 (*strongly disagree*) × number of items per domain × 2 (reviewers)

Overall weighted mean score equation: Calculate a weighted average score using the scaled domain score, number of items per domain and total number of domains:

$$\text{Scaled}\;D1\;x\frac{3}{23}+\text{Scaled}\;D2\;x\frac{3}{23}+\text{Scaled}\;D3\;x\frac{8}{23}+\text{Scaled}\;D4\;x\frac{3}{23}+\text{Scaled}\;D5\;x\frac{4}{23}+\text{Scaled}\;D6\;x\frac{2}{23}$$

**AGREE II scores by domain and mean ‘overall quality’ score**

**Table:**

Paper	Domain 1—Scope and purpose	Domain 2—Stakeholder involvement	Domain 3—Rigour of development	Domain 4—Clarity of presentation	Domain 5—Applicability	Domain 6—Editorial independence	Overall quality score
Stimpfl et al.	75%	25%	45.8%	30.6%	14.6%	95.8%	43.8%
Vinkers et al.	66.7%	16.7%	59.4%	27.8%	0%	91.7%	43.1%

## References

[1] D. Piovani , A. Clavenna , and M. Bonati , “Prescription Prevalence of Psychotropic Drugs in Children and Adolescents: An Analysis of International Data,” European Journal of Clinical Pharmacology 75, no. 10 (2019 Oct): 1333–1346, 10.1007/s00228-019-02711-3. 31270564

[2] National Collaborating Centre for Mental Health (UK) , Violence and Aggression: Short‐Term Management in Mental Health, Health and Community Settings: Updated Edition (British Psychological Society (UK), 2015 (National Institute for Health and Care Excellence: Guidelines)), http://www.ncbi.nlm.nih.gov/books/NBK305020/. 26180871

[3] S. E. Hetrick , J. E. McKenzie , A. P. Bailey , et al., “New Generation Antidepressants for Depression in Children and Adolescents: A Network Meta‐Analysis,” Cochrane Database of Systematic Reviews 2021, no. 5 (2021): CD013674, 10.1002/14651858.CD013674.pub2. PMC8143444 34029378

[4] G. R. Cox , P. Callahan , R. Churchill , et al., “Psychological Therapies Versus Antidepressant Medication, Alone and in Combination for Depression in Children and Adolescents,” Cochrane Database of Systematic Reviews 11 (2012): CD008324, 10.1002/14651858.CD008324.pub2. 23152255

[5] G. M. Kassie , J. Ilomaki , S. J. Wood , et al., “Persistence of Antidepressant Treatment in Children and Adolescents: A Population‐Based Cohort Study,” Australian and New Zealand Journal of Psychiatry 60, no. 7 (2026): 643–652, 10.1177/00048674261418458. 41764082 PMC13291400

[6] F. Movahed , E. Heidari , D. Sadeghi , et al., “Incident Diabetes in Adolescents Using Antidepressant: A Systematic Review and Meta‐Analysis,” European Child & Adolescent Psychiatry 34, no. 2 (2025 Feb): 599–610, 10.1007/s00787-024-02502-x. 38914830

[7] N. Weintrob , D. Cohen , Y. Klipper‐Aurbach , Z. Zadik , and Z. Dickerman , “Decreased Growth During Therapy With Selective Serotonin Reuptake Inhibitors,” Archives of Pediatrics & Adolescent Medicine 156, no. 7 (2002): 696–701, 10.1001/archpedi.156.7.696. 12090838

[8] “Overview | Depression in Children and Young People: Identification and Management | Guidance | NICE,” NICE, (2019), https://www.nice.org.uk/guidance/ng134. 31577402

[9] M. Sandbank , K. Bottema‐Beutel , S. Crowley LaPoint , et al., “Autism Intervention Meta‐Analysis of Early Childhood Studies (Project AIM): Updated Systematic Review and Secondary Analysis,” BMJ 383 (2023): e076733, 10.1136/bmj-2023-076733. 37963634 PMC10644209

[10] E. R. Barnett , A. Z. Trepman , H. A. Fuson , et al., “Deprescribing Psychotropic Medications in Children: Results of a National Qualitative Study,” BMJ Quality and Safety 29, no. 8 (2020): 655–663, 10.1136/bmjqs-2019-010033. 31836627

[11] J. K. Eserian , V. P. Blanco , L. P. Mercuri , J. d. R. Matos , and J. C. F. Galduróz , “Current Strategies for Tapering Psychiatric Drugs: Differing Recommendations, Impractical Doses, and Other Barriers,” Psychiatry Research 329 (2023 Nov): 115537, 10.1016/j.psychres.2023.115537. 37837810

[12] E. Varimo , E. T. Aronen , H. Mogk , H. Rättö , and L. K. Saastamoinen , “Antipsychotic Treatment Duration in Children and Adolescents: A Register‐Based Nationwide Study,” Journal of Child and Adolescent Psychopharmacology 31, no. 6 (2021 Aug): 421–429, 10.1089/cap.2020.0095. 33739863

[13] B. Farrell and D. Mangin , “Deprescribing Is an Essential Part of Good Prescribing,” American Family Physician 99, no. 1 (2019): 7–9. 30600973

[14] D. J. Safer , “Age‐Grouped Differences in Adverse Drug Events From Psychotropic Medication,” Journal of Child and Adolescent Psychopharmacology 21, no. 4 (2011 Aug): 299–309, 10.1089/cap.2010.0152. 21851188

[15] S. C. Dilsaver , “Withdrawal Phenomena Associated With Antidepressant and Antipsychotic Agents,” Drug Safety 10, no. 2 (1994 Feb): 103–114, 10.2165/00002018-199410020-00002. 7912078

[16] M. A. Horowitz , J. E. J. Buckman , R. Saunders , E. Aguirre , J. Davies , and J. Moncrieff , “Antidepressants Withdrawal Effects and Duration of Use: A Survey of Patients Enrolled in Primary Care Psychotherapy Services,” Psychiatry Research 350 (2025 Aug): 116497, 10.1016/j.psychres.2025.116497. 40404538

[17] F. Cosci , V. A. Chouinard , and G. Chouinard , “Discontinuation of Antidepressant Medications: A Significant Healthcare Problem Insufficiently Addressed by the NICE Guidelines,” Psychotherapy and Psychosomatics 92, no. 3 (2023): 148–151, 10.1159/000530692. 37231914

[18] J. Moncrieff , J. Read , and M. A. Horowitz , “The Nature and Impact of Antidepressant Withdrawal Symptoms and Proposal of the Discriminatory Antidepressant Withdrawal Symptoms Scale (DAWSS),” Journal of Affective Disorders Reports 16 (2024): 100765, 10.1016/j.jadr.2024.100765.

[19] M. A. Horowitz and D. Taylor , “Distinguishing Relapse From Antidepressant Withdrawal: Clinical Practice and Antidepressant Discontinuation Studies,” BJPsych Advances 28, no. 5 (2022 Sep): 297–311, 10.1192/bja.2021.62.

[20] M. A. Horowitz and J. Moncrieff , “Gradually Tapering Off Antipsychotics: Lessons for Practice From Case Studies and Neurobiological Principles,” Current Opinion in Psychiatry 37, no. 4 (2024): 320–330, 10.1097/YCO.0000000000000940. 38726815 PMC11139239

[21] M. A. Horowitz , S. Jauhar , S. Natesan , R. M. Murray , and D. Taylor , “A Method for Tapering Antipsychotic Treatment That May Minimize the Risk of Relapse,” Schizophrenia Bulletin 47, no. 4 (2021): 1116–1129, 10.1093/schbul/sbab017. 33754644 PMC8266572

[22] A. Johnston , S. E. Kelly , S. C. Hsieh , B. Skidmore , and G. A. Wells , “Systematic Reviews of Clinical Practice Guidelines: A Methodological Guide,” Journal of Clinical Epidemiology 108 (2019 Apr): 64–76, 10.1016/j.jclinepi.2018.11.030. 30529647

[23] M. J. Page , J. E. McKenzie , P. M. Bossuyt , et al., “The PRISMA 2020 Statement: An Updated Guideline for Reporting Systematic Reviews,” BMJ 372 (2021): n71, 10.1136/bmj.n71. 33782057 PMC8005924

[24] AGREE Next Steps Consortium , “The AGREE II Instrument (Electronic Version),” Canada. [cited 2025 Feb 3], https://www.agreetrust.org/wp‐content/uploads/2017/12/AGREE‐II‐Users‐Manual‐and‐23‐item‐Instrument‐2009‐Update‐2017.pdf.

[25] T. K. Koo and M. Y. Li , “A Guideline of Selecting and Reporting Intraclass Correlation Coefficients for Reliability Research,” Journal of Chiropractic Medicine 15, no. 2 (2016): 155–163, 10.1016/j.jcm.2016.02.012. 27330520 PMC4913118

[26] J. N. Stimpfl , J. T. Walkup , A. S. Robb , et al., “Deprescribing Antidepressants in Children and Adolescents: A Systematic Review of Discontinuation Approaches, Cross‐Titration, and Withdrawal Symptoms,” Journal of Child and Adolescent Psychopharmacology 35, no. 1 (2025): 3–22, 10.1089/cap.2024.0099. 39469761 PMC11971562

[27] C. H. Vinkers , R. W. Kupka , B. W. Penninx , et al., “Discontinuation of Psychotropic Medication: A Synthesis of Evidence Across Medication Classes,” Molecular Psychiatry 29, no. 8 (2024): 2575–2586, 10.1038/s41380-024-02445-4. 38503923 PMC11412909

[28] A. Lerner and M. Klein , “Dependence, Withdrawal and Rebound of CNS Drugs: An Update and Regulatory Considerations for New Drugs Development,” Brain Communications 1, no. 1 (2019): fcz025, 10.1093/braincomms/fcz025. 32954266 PMC7425303

[29] F. Cosci and G. Chouinard , “Acute and Persistent Withdrawal Syndromes Following Discontinuation of Psychotropic Medications,” Psychotherapy and Psychosomatics 89, no. 5 (2020): 283–306, 10.1159/000506868. 32259826

[30] M. A. Horowitz and D. Taylor , “Tapering of SSRI Treatment to Mitigate Withdrawal Symptoms,” Lancet Psychiatry 6, no. 6 (2019 Jun): 538–546, 10.1016/S2215-0366(19)30032-X. 30850328

[31] Y. S. Khan , M. A. S. Khoodoruth , Y. Albobali , and P. M. Haddad , “SSRI Withdrawal Syndrome in Children and Adolescents: A Narrative Literature Review,” Expert Opinion on Drug Safety 22, no. 5 (2023): 381–390, 10.1080/14740338.2023.2224557. 37339264

[32] W. Morgan and M. Baker , “Ensuring Appropriate Use of Psychotropic Medications in Pediatrics: Best Practices in Medication Discontinuation (Deprescribing),” (2024): 63–80, 10.1007/978-3-031-57472-6_4.

[33] C. Gastaldon , G. Schoretsanitis , E. Arzenton , et al., “Withdrawal Syndrome Following Discontinuation of 28 Antidepressants: Pharmacovigilance Analysis of 31,688 Reports From the WHO Spontaneous Reporting Database,” Drug Safety 45, no. 12 (2022): 1539–1549, 10.1007/s40264-022-01246-4. 36400895 PMC9676852

[34] S. Hosenbocus and R. Chahal , “SSRIs and SNRIs: A Review of the Discontinuation Syndrome in Children and Adolescents,” Journal of the Canadian Academy of Child and Adolescent Psychiatry 20, no. 1 (2011): 60–67. 21286371 PMC3024727

[35] M. Horowitz and D. Taylor , The Maudsley Deprescribing Guidelines in Psychiatry: Antidepressants, Benzodiazepines, Gabapentinoids and Z‐drugs (Wiley‐Blackwell, 2024).

[36] P. M. Verbeek‐Heida and E. F. Mathot , “Better Safe Than Sorry—Why Patients Prefer to Stop Using Selective Serotonin Reuptake Inhibitor (SSRI) Antidepressants but Are Afraid to Do So: Results of a Qualitative Study,” Chronic Illness 2, no. 2 (2006 Jun): 133–142, 10.1177/17423953060020020801. 17175656

[37] Depression in Adults: Treatment and Management (National Institute for Health and Care Excellence (NICE), 2022 (National Institute for Health and Care Excellence: Guidelines)), http://www.ncbi.nlm.nih.gov/books/NBK583074/. 35977056

[38] J. van Os and P. C. Groot , “Outcomes of Hyperbolic Tapering of Antidepressants,” Therapeutic Advances in Psychopharmacology 13 (2023): 20451253231171518, 10.1177/20451253231171518. 37200818 PMC10185864

[39] H. K. Batchelor and J. F. Marriott , “Paediatric Pharmacokinetics: Key Considerations,” British Journal of Clinical Pharmacology 79, no. 3 (2015): 395–404, 10.1111/bcp.12267. 25855821 PMC4345950

[40] D. C. Chugani , O. Muzik , C. Juhász , J. J. Janisse , J. Ager , and H. T. Chugani , “Postnatal Maturation of Human GABAA Receptors Measured With Positron Emission Tomography,” Annals of Neurology 49, no. 5 (2001 May): 618–626. 11357952

[41] T. Kendrick , B. Stuart , H. Bowers , et al., Internet and Telephone Intervention to Support Patients Discontinuing Long‐Term Antidepressants in Primary Care: The REDUCE Research Programme Including RCT (National Institute for Health and Care Research, 2025 (Programme Grants for Applied Research)), http://www.ncbi.nlm.nih.gov/books/NBK616349/. 40700552

[42] P. C. Groot and J. van Os , “Outcome of Antidepressant Drug Discontinuation With Taperingstrips After 1‐5 Years,” Therapeutic Advances in Psychopharmacology 10 (2020): 2045125320954609, 10.1177/2045125320954609. 32953040 PMC7476339

[43] C. Bellonci , M. Baker , J. C. Huefner , and R. J. Hilt , “Deprescribing and Its Application to Child Psychiatry,” Child and Adolescent Psychopharmacology News 21, no. 6 (2016 Dec): 1–9, 10.1521/capn.2016.21.6.1.

[44] L. Theall , A. Ninan , and M. Currie , “Findings From an Expert Focus Group on Psychotropic Medication Deprescribing Practices for Children and Youth With Complex Needs,” Frontiers in Child and Adolescent Psychiatry 3 (2024): 1481446, 10.3389/frcha.2024.1481446. 39816603 PMC11731612

[45] D. L. Stutzman , “Long‐Term Use of Antidepressants, Mood Stabilizers, and Antipsychotics in Pediatric Patients With a Focus on Appropriate Deprescribing,” Mental Health Clinician 11, no. 6 (2021): 320–333, 10.9740/mhc.2021.11.320. 34824957 PMC8582767

[46] E. Grudnikoff , K. Fox , and T. G. Lee , “Development of Deprescribing Guidelines in Child and Adolescent Psychiatry: Methodology and Challenges of Systematic Literature Search,” Journal of the American Academy of Child and Adolescent Psychiatry 57, no. 10 (2018): S153–S154, 10.1016/j.jaac.2018.09.071.

[47] L. Sadeghi , “Discontinuing Medication as a Phase of Treatment,” Brown University Child and Adolescent Behavior Letter 33 (2017): 1–6, 10.1002/cbl.30216.

[48] P. Grootens‐Wiegers , I. M. Hein , J. M. van den Broek , and M. C. de Vries , “Medical Decision‐Making in Children and Adolescents: Developmental and Neuroscientific Aspects,” BMC Pediatrics 17 (2017): 120, 10.1186/s12887-017-0869-x. 28482854 PMC5422908

[49] J. K. Eserian , F. F. de Oliveira , L. P. Mercuri , J. D. R. Matos , and J. C. F. Galduróz , “Experimental Evaluation of Guideline‐Recommended Pharmaceutical Manipulations for Hyperbolic Tapering of Psychiatric Drugs,” British Journal of Psychiatry (2026): 1–8, 10.1192/bjp.2026.10663. 42152557

[50] F. Bird , J. M. Harper , J. K. Luiselli , A. Shlesinger , and J. Gold , “Psychotropic Medication Monitoring in a Human Services Organization for Children With Autism Spectrum Disorder: Description and Evaluation of Interdisciplinary Team Review,” Behavior Analysis in Practice 15, no. 4 (2022 Dec): 1337–1347, 10.1007/s40617-022-00699-4. 35371414 PMC8956327

[51] D. Adams , R. P. Hastings , I. Maidment , C. Shah , and P. E. Langdon , “Deprescribing Psychotropic Medicines for Behaviours That Challenge in People With Intellectual Disabilities: A Systematic Review,” BMC Psychiatry 23, no. 1 (2023): 202, 10.1186/s12888-022-04479-w. 36978032 PMC10044393

[52] I. Bužančić , I. Kummer , M. Držaić , and M. Ortner Hadžiabdić , “Community‐Based Pharmacists' Role in Deprescribing: A Systematic Review,” British Journal of Clinical Pharmacology 88, no. 2 (2022): 452–463, 10.1111/bcp.14947. 34155673

[53] R. E. Cooper , M. Ashman , J. Lomani , et al., “‘Stabilise‐Reduce, Stabilise‐Reduce’: A Survey of the Common Practices of Deprescribing Services and Recommendations for Future Services,” PLoS ONE 18, no. 3 (2023): e0282988, 10.1371/journal.pone.0282988. 36920968 PMC10016688

